# A microarray analysis of full depth knee cartilage of ovariectomized rats

**DOI:** 10.1186/1756-0500-4-63

**Published:** 2011-03-15

**Authors:** Anne C Bay-Jensen, Rasmus H Nielsen, Toni Segovia-Silvestre, Moïse Azria, Frank Staedtler, Martin Letzkus, Nicole Hartmann, Arndt H Brachat, Morten A Karsdal

**Affiliations:** 1 Cartilage biology and biomarkers, Nordic Bioscience, Herlev, Denmark; 2 Novartis Institutes for BioMedical Research, Novartis Pharma AG, Basel, Switzerland

## Abstract

**Background:**

This short communication focuses the on articular cartilage and the subchondral bone, both of which play important roles in the development of osteoarthritis (OA). There are indications that estrogen-deficiency, as the post-menopausal state, accelerate the development of OA.

**Findings:**

We investigated, which extracellular matrix (ECM) protein, proteases and different pro-inflammatory factors was up- or down-regulated in the knee joint tissue in response to estrogen-deficiency in rats induced by ovariectomy. These data support previous findings that several metalloproteinases (MMPs) and cysteine proteases are co-regulated with numerous collagens and proteoglycans that are important for cartilage integrity. Furthermore quite a few pro-inflammatory cytokines were regulated by estrogen deprivation.

**Conclusion:**

We found multiple genes where regulated in the joint by estrogen-deficiency, many of which correspond well with our current knowledge of the pathogenesis of OA. It supports that estrogen-deficiency (e.g. OVX) may accelerate joint deterioration. However, there are also data that draw attention the need for better understanding of the synergy between proteases and tissue turnover.

## Introduction

Post-menopausal women have a higher incident rate of osteoarthritis (OA) and osteoporosis (OP) than that of age-matched men [1,2]. There are indications that peri-menopausal women receiving estrogen replacement therapy (ERT) had lower risk of developing radiographic knee and hip OA and that the protective effect was increased with increased duration of ERT treatment [1]. This is in line with findings that also bone turnover is increased due to estrogen-deficiency [3]. Mouritzen et al. (2005) found that urinary levels of cartilage and bone turnover markers (i.e. C-terminal telopeptide of type I and II collagen, CTX-I/II) were increased in post-menopausal women compared to pre-menopausal women. Since then it has been shown that selective estrogen-receptor modulators (SERMs) given to postmenopausal women decrease the level of CTX-II about 50% of baseline (12-month follow-up). These markers are generated by enzymatic processing of type II and I collagen, respectively, which is a result of induced cellular responses. Furthermore, it has been shown that adult articular cartilage expresses estrogen receptors and these can be activated and thereby induce production of extracellular matrix (ECM) molecules (e.g. proteoglycans and collagens) [4]. Ovariectomy of rats led to similar results; estrogen-deficiency led to cartilage damage and increased bone resorption, which could be prevented by administrations of estrogen [5^-^7]. Comparable studies have been done in other species [8]. We investigated which genes related to cartilage turnover and integrity was regulated in response to estrogen deprivation by comparing the mRNA expression between sham and ovariectomized rats.

## Methods

**Figure 1:**
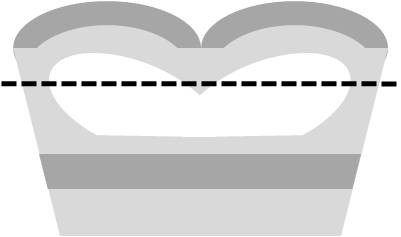
**The tibia plateau was separated from the tibia by a cut (broken line) in the upper half of the secondary ossification site (white area)**. The light gray of the cut-off piece depicts the subchondral bone and the dark grey the articular cartilage. Total RNA was extracted from this piece.

Six month old female rats were either ovariectomized (OVX, n = 5) or sham operated (n = 5). After recovery the rats were house under standard conditions for 8 weeks, and terminated. All animal experiments were approved by the local ethical authorities ("Dyreforsøgstilsynet", approval no. DK-2006/561-1239). The knees were isolated right after termination and the cartilage and subchondral compartment was detached from the tibia and flash frozen in liquid nitrogen (Figure 1). Total RNA was isolated from the pulverized tissue using standard phenol based extraction method. The mRNA was hybridized to an Affymetrix GeneChip [9].

Expression data was retrieved from the internal microarray database discriminating 1.5-fold and 2-fold significant (p < 0.05 by Student's t-test) increased or decreased expression of individual genes when comparing the OVX and sham group, which gave a list of 2.959 and 579 genes, respectively. An objective search where made on database terms: Extracellular matrix protein, Protease/Proteinase, Cytokine, Growth factor, Bone and Cartilage. From these searched, literature based and subjectively guided sorting was done on structural proteins involved in cartilage and bone tissue remodeling and destruction, as well as cartilage and bone related factors. Each of the genes in question was reviewed by searches (term: "gene name" cartilage and/or bone) on Pubmed http://www.ncbi.nlm.nih.gov/pubmed/, IHOP http://www.ihop-net.org/UniPub/iHOP/, Rat genome database http://rgd.mcw.edu/tools/genes/ and HUGO gene nomenclature Committee http://www.genenames.org/.

### Expression of ECM proteins in response to estrogen deficiency

**Figure 2:**
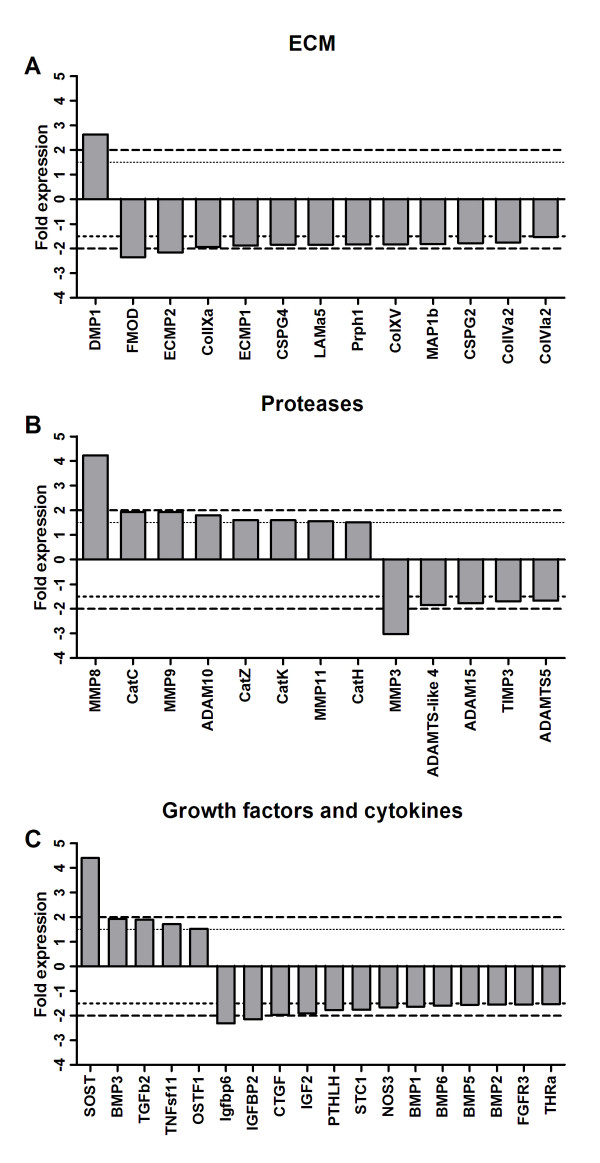
**Comparison between the expression levels of selective genes in the tibia plateau of OVX rats and sham rats (indicated as fold expression of sham)**. A) The fold expression of ECM proteins; B) The fold expression of ECM proteases and; and, C) The fold expression of growth factors and cytokines involved in cartilage and bone turnover. Dotted line indicates a 1.5-fold expression level compared to sham. P < 0.05 were for all.

A total of nine relevant ECM proteins genes were found to be differentially expressed in the OVX group compared to the sham group (Figure 2A). Only one ECM gene were significantly up-regulated; Dentin matrix protein 1 (DMP1). DMP1 is expressed in mineralized tissues such as hypertrophic cartilage and bone, where it can bind Ca^2+ ^and regulate matrix mineralization [10]. There were eight ECM genes down-regulated. Fibromodulin (FMOD) is known to interact with both type I and II collagen in bone and cartilage, respectively [11]. Through its binding of collagens it aids stabilization of the tissue. Several collagens (Col) were down-regulated; type IVa2, VIa2, IXa and XV. Type VI and IX are some of the minor collagens of the mature cartilage that are known to interact with other matrix components, such as hyaluronan, heperan sulfate and Chondroitin sulfate proteoglycans. Type XV collagen is member of the FACIT collagen family (fibril-associated collagens with interrupted helices) and mainly expressed by fibroblastic cells [12]. Type IV collagen, is a collagen of the basal lamina and widely distributed throughout the body, like many other collagens it is involved in tissue stabilization and flexibility [12]. Two chondroitin sulfate proglycans were also down-regulated in the joint tissue of OVX rats: CSPG2 (Versican) and 4 (NG2) (Figure 2A). Both have been shown to be involved in the regulations of bone development, chondrogenesis and angiogenesis, but only present in low amounts in mature cartilage and bone [13]. Laminin alpha 5 (LAMa5), a subunit of laminin 5, which is observed in the basement membrane. It has been shown to promote bone growth and regeneration through cell adhesion and motility [14].

### Expression of ECM degrading proteases in response to estrogen deficiency

Eight ECM degrading proteases were up-regulated in the joints of the OVX rats (Figure 2B). Metalloproteinase (MMP) 8 (collagenase-2), 9 (gelatinase B), 11 (stromelysin-3) and 16 (MT3-MMP) are collagen and proteoglycan degrading protease, which have been implicated as drivers of joint degradation [15]. Cathepsins (Cat) C, Z, K and H, which are all cysteine proteases, have been shown to play vital role the regulation of tissue turnover. CatC is a dipeptidyl aminopeptidase, which is involved in the activation of serine proteases such as CatG and elastase [16]. CatZ is also a dipeptidyl peptidase, but its function in cartilage and bone turnover is unclear. CatK is mainly expressed by osteoclast and are responsible for the release of the bone turnover marker CTX-I. It has been found to be elevated in OVX animals and post-menopausal women. CatH cleaves osteocalcin in bone and might therefore be an important protease in bone turnover [17]. The A Disintegrin And Metallopeptidase 10 (ADAM10) is expressed by chondrocyte in bone development and in OA chondrocytes. It is therefore believed to be involved in the remodeling of cartilage [18].

Six proteases were down-regulated; MMP3 and the aggrecanases ADAMTS-like 4, ADAM15 and ADAMTS5, as well as bone morphogenic protein (BMP) 1 and tissue inhibitor metalloproteinase 3 (TIMP3) (Figure 2B). MMP3 can degrade fibrillar collagens such as type II and I collagen [19]. MMP3 has been shown to be elevated in inflammatory diseases, such as rheumatoid arthritis; however it is mainly expressed by the connective tissue like the synovial membrane and to a lesser degree by bone and cartilage. ADAM15 has been shown to cleave ECM components such as gelatin and type IV collagen. It is has been found to be upregrulated in OA chondrocytes [20]. ADAMTS-like 4 and ADAMTS5 (ADAM with thrombin mortif) are known for their capability to degrade proteoglycans such as aggrecan and versican [21]. It intriguing that ADAMTS5 is down-regulated, since previous studies have indicated it to be a primary protease in OA cartilage degradation [22]. In contrast, others have shown ADAMTS' are involved in both degradation and remodeling of the tissue [23,24]. The endogenous MMP and aggrecanase inhibitor TIMP3 was also down-regulated. TIMP3 is believed to be an important regulator of cartilage and bone turnover, through its inhibition of MMPs and aggrecanases [21]. In contrast to other BMPs, BMP1 does not belong to the TGF-β family of proteins. It is a metalloproteinase that acts on procollagen I, II, and III and has been implicated in cartilage development [25].

### Expression of cytokines and growth factors in response to estrogen deficiency

Next we searched for factors directly involved in bone and/or cartilage regulation. We found that five genes were significantly up-regulated and 11 were significantly down-regulated in the OVX animals (Figure 2C). Sclerostin (SOST) was markedly over-expressed in the OVX rats. It is expressed by osteoclast and is a negative regulator of bone formation through activation of the Wnt pathway [26]. BMP2 through BMP7 belong to the transforming growth factor beta superfamily of proteins. Whereas BMP6, -5 and -2 were down-regulated, BMP3 was up-regulated. BMP3 is known to induce bone formation and cartilage development and to have antagonistic effect on other BMPs [27]. Transforming growth factors beta-2 (TGFb2) expression was also elevated. TGFb2 is a multi-factorial regulator of cellular growth in developing systems and in repair of adult bone and cartilage [28]. TNFsf11, also known as RANKL, is a natural and necessary surface-bound molecule that activates osteoclasts, cells involved in bone resorption. Overproduction of RANKL is implicated in a variety of degenerative bone diseases, such as arthritis [29]. Osteoclast stimulating factor 1 (OSTF1) expression was also increased in the OVX rats' joints (Figure 2C). It is known to increase osteoclast formation and bone resorption through signal transduction, however only little is known about the family member of osteoblast stimulation factors [30]. Insulin-like growth factor 2 (IGF2), IGF binding protein 2 (IGFBP) and IGFBP6 were all down-regulated in the OVX rats. IGF2 is known to bind both to IGFBP2 and IGFBP6, and its effect on bone cells is thereby prevented (e.g. osteoblast proliferation) [31]. Connective tissue growth factor (CTGF) is a potent stimulator for proliferation and differentiation of osteoblast and chondrocytes through regulation of cellular associated genes [32]. The tightly related proteins Fibroblast growth factor receptor 3 (FGFR3) and the parathyroid hormone-like hormone (PTHLH), which were both down-regulated, play central roles in the physiological regulation of bone formation, by promoting recruitment and survival of osteoblasts, and probably plays a role in the physiological regulation of bone resorption, by enhancing osteoclast formation. Signaling by FGFR3 and PTHLH coordinates cartilage and bone development. PTHLH is also an essential physiological regulator of adult bone mass [33]. The calcitropic hormone Stanniocalcin 1 (STC1) was down-regulated in the OVX rats. It is involved in the regulation of longitudinal bone growth [34]. Nitric oxide synthase 3 (NOS3, eNOS) was also down-regulated. It has been shown that estrogen induces NOS3 expression in endothelial cells and osteoblasts, raising the possibility that NO derived from the NOS3 pathway plays a role in mediating the effects of sex hormones on bone [35]. BMP2, -5 and -6, which were down-regulated in the OVX rats (Figure 2C), all induces cartilage and bone formation and play role in joint integrity [27].

## Discussion and Conclusion

We were interested in investigating the effect of ovariectomy (i.e. estrogen deficiency) on the joint. Here we use a rodent model that in previous study has been shown to develop bone and cartilage changes, resembling pathological features of OA. We have made a guided search on ECM molecules, matrix proteases and cytokines/growth factors that have previously been directly implicated in bone and cartilage turnover. Although the search was made electronically, it is still somewhat subjective, because the database keywords may not be ubiquitous and fulfilling. Nevertheless we found several differentially express genes, which are known to play a role in development of joint degenerative disease such as OA.

Animal studies have shown that by inhibiting osteoclast activity, the histo-pathological score (cf. Mankin) could be markedly reduced, suggesting that osteoclasts play a central role in OA and cartilage breakdown. Also bone formation cells, osteoblast, seem to play an important role in OA: osteoblasts isolated from subchondral bone of OA hips demonstrate an altered and more active phenotype. Furthermore, IGF-1 (a bone promoting factor as well as several growth factors (e.g. BMPs, TGF and FGF) has been shown to be elevated in OA-subchondral explant cultures.

In conclusion, we found that extracellular matrix proteins, proteases and paracrine factors known to be involved in joint degenerative diseases were either down- or up-regulated in response to estrogen-depletion. As a whole, the data support current knowledge; however there are some discrepancies, which stresses that more investigation is needed to fully understand the pathogenesis of post-menopausal OA.

## Competing interests

The authors declare that they have no competing interests.

## Authors' contributions

Conception and design: RHN, MAK, AB. Analysis and interpretation of the data: ACBJ. Drafting of the article: ACBJ. Critical revision of the article for important intellectual content, MAK, MA, NH, FS and ML. Final approval of the article: MAK. Provision of study materials or patients: N/A. Statistical expertise: AB. Obtaining of funding: MAK. Administrative, technical, or logistic support: N/A. Collection and assembly of data: ACBJ. All authors have approved this final version of the manuscript.
